# Multitarget Effects of Danqi Pill on Global Gene Expression Changes in Myocardial Ischemia

**DOI:** 10.1155/2018/9469670

**Published:** 2018-02-01

**Authors:** Qiyan Wang, Hui Meng, Qian Zhang, Tianjiao Shi, Xuefeng Zhang, Mingyan Shao, Linghui Lu, Jing Wang, Wei Wang, Chun Li, Yong Wang

**Affiliations:** ^1^School of Life Sciences, Beijing University of Chinese Medicine, Beijing 100029, China; ^2^Modern Research Center for Traditional Chinese Medicine, Beijing University of Chinese Medicine, Beijing 100029, China; ^3^School of Chinese Medicine, Beijing University of Chinese Medicine, Beijing 100029, China; ^4^Staidson (Beijing) Biopharmaceuticals Co., Ltd., Beijing 100176, China

## Abstract

Danqi pill (DQP) is a widely prescribed traditional Chinese medicine (TCM) in the treatment of cardiovascular diseases. The objective of this study is to systematically characterize altered gene expression pattern induced by myocardial ischemia (MI) in a rat model and to investigate the effects of DQP on global gene expression. Global mRNA expression was measured. Differentially expressed genes among the sham group, model group, and DQP group were analyzed. The gene ontology enrichment analysis and pathway analysis of differentially expressed genes were carried out. We quantified 10,813 genes. Compared with the sham group, expressions of 339 genes were upregulated and 177 genes were downregulated in the model group. The upregulated genes were enriched in extracellular matrix organization, response to wounding, and defense response pathways. Downregulated genes were enriched in fatty acid metabolism, pyruvate metabolism, PPAR signaling pathways, and so forth. This indicated that energy metabolic disorders occurred in rats with MI. In the DQP group, expressions of genes in the altered pathways were regulated back towards normal levels. DQP reversed expression of 313 of the 516 differentially expressed genes in the model group. This study provides insight into the multitarget mechanism of TCM in the treatment of complex diseases.

## 1. Introduction

Acute myocardial ischemia (MI) occurs when blood flow stops to a part of the heart causing damage to the myocardial tissue, and MI is one of the leading causes of death worldwide [1–3]. How to reduce MI-caused mortality is a major challenge to the entire medical community. Conventional managements of MI include intravenous thrombolysis, percutaneous coronary intervention, optimization of oxygenation, and pain control [4]. Danqi pill (DQP), composed of *Salvia miltiorrhiza* Bunge and *Panax notoginseng*, is a widely prescribed traditional Chinese medicine (TCM) in the treatment of a variety of cardiovascular diseases [5, 6]. DQP has been used as an alternative or complementary medicine in the prevention and treatment of MI in China. There are multiple components of DQP that have potential regulative effects on multiple targets in the treatment of cardiovascular diseases [7–10]. However, the overall regulative effect of DQP has not been explored yet. Investigations into the effects of DQP on global gene expressions will further our understanding on the mechanisms of TCM in treating complex diseases.

In recent years, transcriptome sequencing technologies have been developing very fast. Digital Gene Expression (DGE) technique is one of the RNA-sequencing methods for the analysis of differentially expressed genes in different samples [11]. DGE has more accuracy in quantification of gene expression levels compared with microarray technologies. It is able to provide quantitative readout of mRNA expression levels in samples [12]. Illumina's sequencing platform was applied in our present study.

Acute MI model was induced by ligation of left anterior descending (LAD) coronary artery in rats. The rats were divided into three groups: sham-operated group, model group, and DQP-treated group. Gene expressions in the three groups were measured by RNA-sequencing technology, and differentially expressed genes and signaling pathways among different groups were analyzed. This study will expand our knowledge on the multitarget mechanism of DQP in the treatment of ischemic heart disease.

## 2. Materials and Methods

### 2.1. Grouping of Animals

Sprague-Dawley (SD) male rats, weighing 220 ± 10 g, were randomly divided into three groups: sham-operated group, acute MI model group, and DQP-treated group. Each group contained 10 rats. The animals were purchased from Beijing Vital River Laboratory Animal Technology Co., Ltd. This study complied with the China Physiological Society's “Guiding Principles in the Care and Use of Animal” and got the approval of Animal Care Committee of Beijing University of Chinese Medicine.

MI model was induced in model and DQP group by direct LAD artery ligation as previously described [13, 14]. Briefly, SD rats were anaesthetized intraperitoneally with pentobarbital sodium (1%, 50 mg/kg). The left anterior descending coronary artery was ligated proximal to its main branching point with a 5–0 polypropylene suture. Sham-operated rats went through identical thoracotomy procedure but their coronary arteries were not ligated. After the operations, all of the rats were fed with a standard diet and were maintained on a 12 h light and dark cycle for 28 days. Animals in the DQP group were treated with concentrated DQP (purchased from Tongren Tang, Beijing, China) dissolved in pure water, with the daily dosage of 1.5 g/kg, for 28 consecutive days beginning from the day after the operation. At the end of the study, all rats were anaesthetized using pentobarbital sodium following an overnight fast. Rats were sacrificed and the left ventricle was carefully dissected to keep only the viable myocardium in the marginal zone of the infarct region in the DQP group and MI model group. The same region in the sham group was also dissected. Dissected heart tissues were frozen and stored in a freezer at −80°C before RNA extraction.

### 2.2. Echocardiographic Assessment of Heart Function

Echocardiography (Vevo 2100, Visual Sonics, Canada) was applied to assess the cardiac function-related parameters, including left ventricular ejection fraction (LVEF), left ventricular internal diameter at end-diastole (LVID;d) and at end-systole (LVID;s), and left ventricular fractional shortening (LVFS). LVEF and LVFS were calculated automatically by the software.

### 2.3. RNA Preparation

Heart tissues of the 3 rats in each group were homogenized in liquid nitrogen for RNA sequencing. Total RNA of the heart tissues were extracted using TRIzol Reagent® (Invitrogen, Carlsbad, CA), following the instruction of the manufacturer. Extracted RNA was treated with DNase to remove potential genomic DNA contamination. The quality of RNA was examined by Agilent 2100 Bioanalyzer (Agilent Technologies, Palo Alto, CA, USA).

### 2.4. cDNA Library Preparation and RNA Sequencing

Sequence tag was prepared using Illumina's Digital Gene Expression Tag Profiling Kit according to the manufacturer's protocol. Briefly, mRNA was purified from total RNA by binding mRNA to a magnetic oligo(dT) bead. Oligo(dT) was then used as a primer to synthesize cDNA. The bead-bound cDNA was subsequently digested with restriction enzyme NlaIII, which recognizes and cuts off the CATG sites. The fragments apart from the 3′ cDNA fragments connected to oligo(dT) beads are washed away and the Illumina adaptor 1 is ligated to the sticky 5′ end of the digested bead-bound cDNA fragments. Mme I, a type of endonuclease with separated recognition sites and digestion sites, was used to cut at 17 bp downstream of the CATG site, producing tags with adaptor 1. After removing 3′ fragments with magnetic beads precipitation, Illumina adaptor 2 is ligated to the 3′ ends of tags. A tag library with different adaptors of both ends was thus formed.

After PCR amplification of 12 cycles, fragments were purified by a 6% Novex TBE PAGE Gel electrophoresis. In the end, the purified cDNA tags were sequenced using Illumina HiSeq™ 2000 at BGI-Shenzhen.

### 2.5. Analysis of Sequencing Data and Tag Mapping

During the quality control steps, Agilent 2100 Bioanalyzer was used in quantification and qualification of the sample library. Low-quality tags and tags of copy number less than two were filtered to produce clean tags. The clean tags were classified according to their copy number in the library. Clean tags were aligned to the rat reference sequences and annotated. Clean tag numbers corresponding to each gene were counted in each sample.

### 2.6. Analysis of Differentially Expressed Genes

The number of clean tags in each sample was normalized to transcripts per million (TPM) and TPM values were used to calculate fold change and false discovery rate across different groups. To avoid possible noise signal from high-throughput sequencing, the genes with TPM less than 3 in 2 or more samples were excluded. Analysis of differentially expressed genes among different groups was performed in R (version 3.0.2) with edgeR Bioconductor package [15]. EdgeR uses empirical Bayes estimation and exact tests based on the negative binomial distribution. The resulting *P* values for all genes were corrected for multiple tests using a FDR adjustment. In this study, the fold change larger than 2 and FDR less than 0.01 were used to define the differentially expressed gene.

### 2.7. Gene Functional and Enrichment Analysis

The gene ontology (GO) enrichment analysis and Kyoto Encyclopedia of Genes and Genomes (KEGG) pathway analysis of all differentially expressed genes were carried out using the DAVID Functional Annotation Tool [16]. According to the transcription factor database (TFdb) (http://genome.gsc.riken.jp/TFdb/), transcription factors that had altered expressions induced by ischemia were selected. The target genes of these transcription factors were searched through databases, including Transcriptional Regulatory Element Database (http://rulai.cshl.edu/TRED), Human Transcriptional Regulation interaction database (http://www.lbbc.ibb.unesp.br/htri/index.jsp), and Transcription factor checkpoint (http://www.tfcheckpoint.org/index.php/search) [17]. Regulation networks were constructed using Cytoscape software [18].

### 2.8. Messenger RNA Expression by Quantitative Real-Time PCR

Real-time PCR was applied to validate differentially expressed genes in six samples in each group. First-strand cDNA was synthesized from total RNA with a RevertAid First Strand cDNA Synthesis Kit (Thermo Scientific, USA, lot number: K1622) according to the manufacturer's instruction. Quantitative real-time PCR assays were performed using C1000 Thermal Cycler PCR machine (Bio-Rad, USA). The reaction volume was 20 *μ*l including 1 *μ*l forward and reverse primer pairs, 2 *μ*l cDNA, 10 *μ*l FastStart Universal SYBR Green Master (Roche, Germany, lot number: 04913914001), and 7 *μ*l RNase free water. The PCR procedures were as follows: 15 s at 95°C for denaturation and 1 min at 60°C for annealing and extension. Ct values were obtained after 40 cycles of reactions. Primer sequences of each gene were listed in Table 1. Ct values of targeted mRNA were normalized to the Ct values of GAPDH. Relative expressions of these genes were calculated by the 2^−ΔΔCT^ method.

## 3. Results

### 3.1. Effects of DQP on Cardiac Function

**Figure 1:**
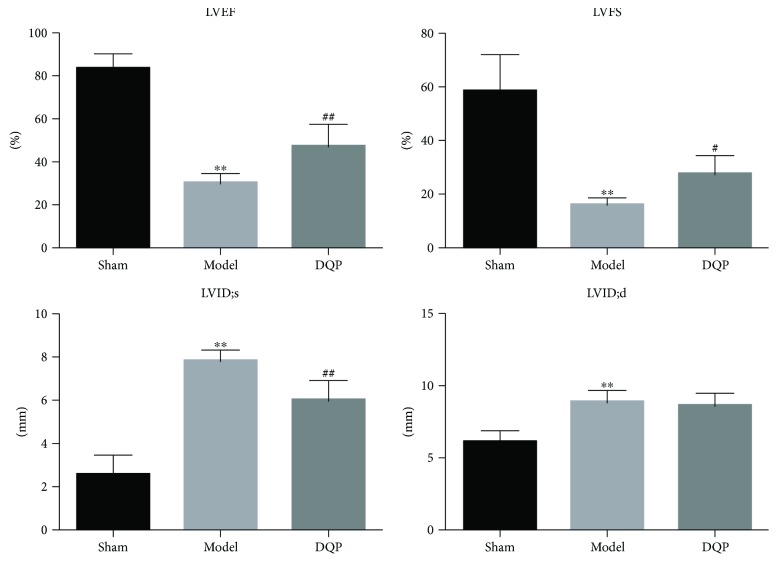
Indicators of heart functions in three groups of rats. LVEF, LVFS, LVID;s, and LVID;d in the model group were significantly changed compared with those in the sham group (*N* = 10 in each group, *P* < 0.01). In the DQP-treated group, LVEF and LVFS were significantly upregulated compared with the model group (*P* < 0.05). LVID;d and LVID;s in the DQP-treated group were also downregulated, though the difference of LVID;d was not statistically significant. ^∗∗^*P* < 0.01 versus sham group; ^#^*P* < 0.05 versus model group; ^##^*P* < 0.01 versus model group.

Twenty-eight days after surgery, echocardiography showed that LVEF and LVFS of rats that underwent ligation in model group were downregulated significantly by 63.7% and 72.7%, compared with those of the sham group (Figure 1, *P* < 0.01), indicating that cardiac function of rats in the model group was impaired and a MI model was established. LVID;s and LVID;d increased by 204.9% and 44.3% in the model group compared with those in the sham group. After treatment with DQP, the LVEF and LVFS were upregulated by 56.3% and 72.1%, compared with those in the model group (*P* < 0.05). LVID;s and LVID;d also decreased by 23.3% and 2.4% after treatment with DQP, suggesting that DQP could improve cardiac functions in the MI model (Figure 1).

### 3.2. High-Throughput Sequencing Data and Differentially Expressed Genes

In total, we obtained four million raw tags and over 3.7 high-quality clean tags from each sample. 51.8%, 47.8%, and 52.5% tags were mapped to annotated rat genomes in the DQP-treated, sham-operated, and model rats, respectively. About 5% of the tags were unknown ones. Altogether, 10,813 genes were detected, and 9537, 8907, and 9344 genes were detected and quantified in the DQP-treated, sham-operated, and model rats, respectively (Supplement Table 1).

Redundancy and heterogeneity are characteristics of mRNA expression. The majority of mRNAs have low expression level, whereas a minority of mRNAs has high abundance of expression. In this study, among the distinct clean tags in the nine libraries, the majority of distinct clean tags (60.21%–63.31%) had 2–5 copies. Only 3.54%–4.47% clean tags had more than 100 copies. The distribution of distinct clean tags showed a similar tendency, indicating that gene expression patterns among the libraries were similar.

Differentially expressed genes were analyzed using the following criteria: fold change > 2 and FDR < 0.01. The analysis showed that compared with the sham-operated group, 339 genes were upregulated and 177 genes were downregulated significantly in the model group, demonstrating that gene expression pattern was altered under ischemic condition. Compared with the model group, 886 genes were upregulated and 1082 genes were downregulated in the DQP-treated group.

**Figure 2:**
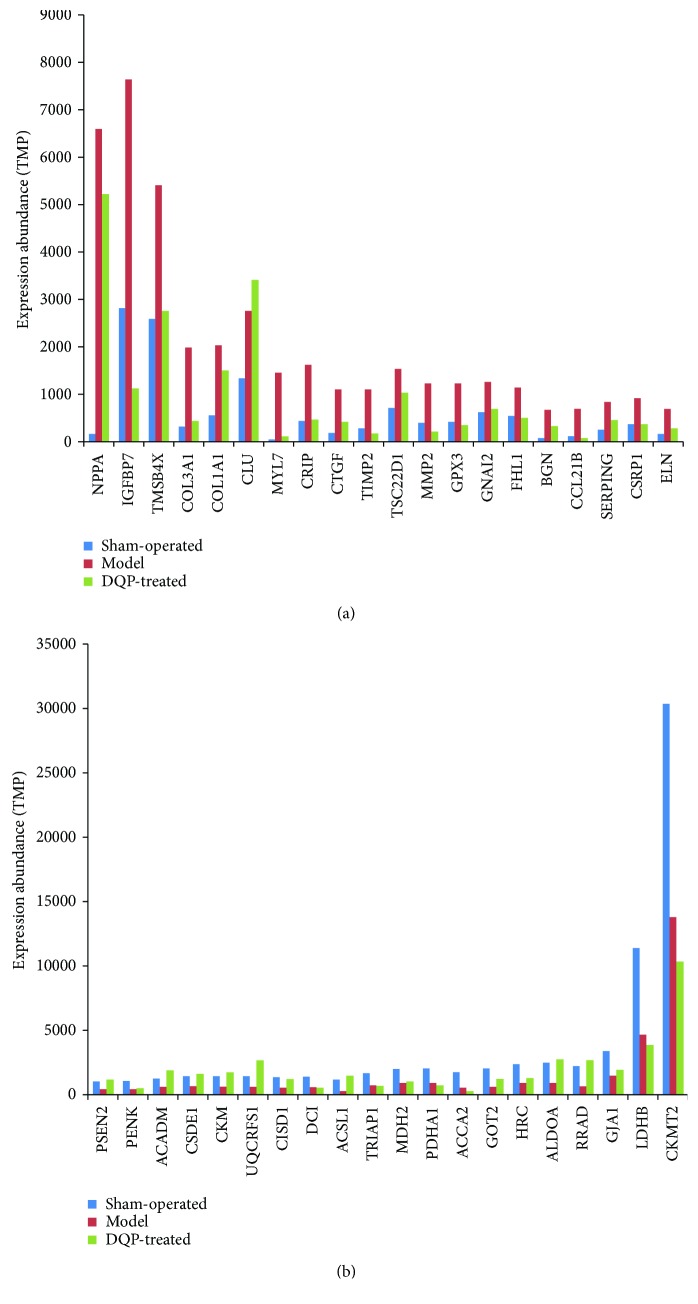
Expression abundance changes. (a) Top 20 genes upregulated in the model group compared with the sham-operated group and their expression abundances in the DQP-treated group. (b) Top 20 genes downregulated in the model group and their expression abundances in the DQP-treated group.

Genes were expressed at different abundances, and expressions of some genes were altered remarkably in ischemic heart tissues. The top 20 upregulated and 20 downregulated genes in regard to abundance in the model compared with the sham-operated group and their expressions in DQP group were shown in Figure 2. NPPA, which encodes atrial natriuretic factor precursor, was upregulated greatly in ischemic heart. Insulin-like growth factor binding protein 7 (IGFBP7) was also upregulated. Other genes that were upregulated in great abundance encode collagen (COL3A1, COL1A1), light chain of myosin (MYL7), metallopeptidase (MMP2), and so forth. The top 20 downregulated genes in the model group are involved in fatty acid metabolism (ACAA2, ACSL1, ACADM, and DCI) and glycolysis (ALDOA, LDHB, MDH2, and PDHA1). Most of the products of the downregulated genes are located in the mitochondria. Among the top 20 genes that were upregulated or downregulated in the model group, DQP was able to regulate the expression of 19 and 14 genes back towards normal levels, respectively (Figure 2).

### 3.3. Biological Pathways and Processes Affected in Ischemia Heart

To investigate the global gene expression changes in ischemic heart in the model group, we studied the biological pathways and processes affected in ischemic heart by KEGG pathway enrichment and GO enrichment analysis. The upregulated KEGG pathways and biological processes mainly include complement and coagulation cascades, ECM-receptor interaction, dilated cardiomyopathy, response to wounding, coagulation, immune response, extracellular matrix organization, focal adhesion, skeletal system development, and cell growth (Tables 2 and 3). The downregulated biological processes and pathways mainly involve in nutrient metabolism and energy supply, such as fatty acid metabolism, pyruvate metabolism, amino acid metabolism, carbohydrate catabolism, citrate cycle, and oxidation reduction (Tables 2 and 3). This analysis illustrated that the energy supply of the heart was seriously compromised and the myocardial remodeling took place to compensate for the short supply of energy in the ischemic process.

### 3.4. Genes and Signaling Pathways Regulated by DQP

Expressions of 516 genes were significantly affected by MI. In the DQP-treated group, expressions of 313 out of these 516 genes were reversed by DQP. These overlapped genes are considered as DQP-responsive genes. Of the 339 genes that were upregulated in the model group, 212 were significantly downregulated in the DQP treatment group. These genes were enriched in infection and immunity, cell growth, extracellular matrix deposition, and so forth (Table 4). 46% of proteins encoded by the downregulated genes were located in extracellular space. Among the 177 downregulated genes in the model group, DQP significantly reversed expressions of 101 genes. The enriched pathways and biological processes were involved in energy embolisms, such as carbon metabolism, fatty acid degradation, and pyruvate metabolism (Table 5). 25% of the proteins encoded by the downregulated genes were located in the mitochondrion. These results demonstrated that DQP can regulate multiple pathways altered under ischemic stimulus.

### 3.5. Transcriptional Regulations by DQP

**Figure 3:**
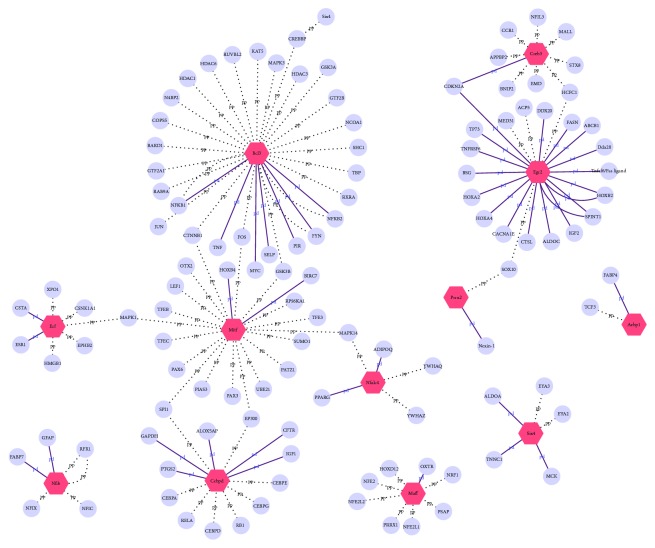
Regulative network by altered transcription factors. Transcription factors were represented as red octagons. Regulated proteins were represented as blue circles. Dot lines represented protein-protein interaction, and solid lines represented protein-gene interactions.

Transcription factors play critical roles in regulating expressions of target genes. According to the transcription database, we searched transcription factors that had altered expressions induced by ischemic stimuli. Among the dysregulated 516 genes in the MI model group, 12 were transcription factors, including CEBPD, CREB3, BCL3, SIX4, NFATC4, MAFF, ERF, NFIB, MITF, AEBP1, EGR2, and PRRX2. The target genes of these transcription factors were also searched out through databases, and the regulative networks were constructed (Figure 3). Expressions of SIX4, NFIB, and MITF were downregulated in the model group compared with the sham-operated group and the expressions of the nine genes were downregulated. The deregulated transcription factors play roles in inflammation, lipid metabolism, mitochondrial metabolism, and cardiac fibrosis. DQP treatment regulated expressions of the 12 transcription factors towards normal levels.

### 3.6. Real-Time PCR Validation Analyses

**Figure 4:**
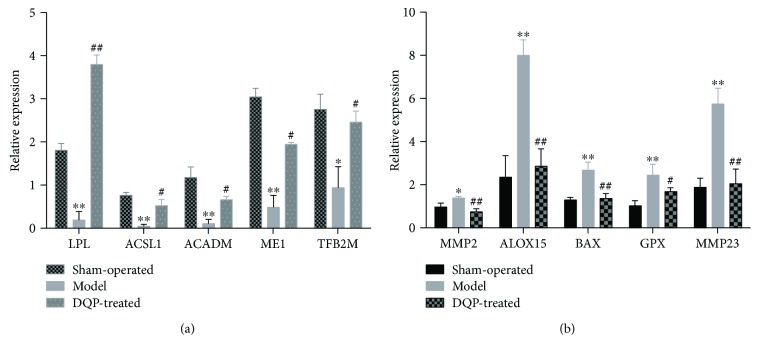
Validations of expressions of genes by real-time PCR. (a) Expressions of five genes involved in fatty acids were downregulated in the model group and upregulated by DQP treatment. (b) Expressions of five genes were increased in the MI model group and reduced by DQP treatment. Each group contained six samples. ^∗^*P* < 0.05 versus sham group; ^∗∗^*P* < 0.01 versus sham group; ^#^*P* < 0.05 versus model group; ^##^*P* < 0.01 versus model group.

To validate the RNA-sequencing results, we measured the expression patterns of 10 genes by quantitative real-time PCR analyses. Five genes involved in fatty acid metabolism pathways were chosen for validations. Real-time PCR results showed that expressions of all of these five genes were downregulated in the model group and upregulated by DQP treatment, consistent with RNA-sequencing results (Figure 4(a)). Expressions of another five genes involved in ventricular remodeling, cellular apoptosis, and inflammation were also validated. Real-time PCP results showed that expressions of these five genes were upregulated in the model group and downregulated by DQP treatment, which were also consistent with RNA-sequencing results (Figure 4(b)).

## 4. Discussion

MI is one of the cardiovascular conditions that threaten people's health, and TCM has been shown to be effective in attenuating symptoms of MI. The aim of this study was to systematically analyze the effects of DQP on altered global gene expression patterns induced by MI. An MI rat model was induced by coronary artery ligation, and Illumina's RNA-seq platform was applied in this study. The results showed that heart functions were impaired in the MI model rat and DQP treatment protected heart functions at 28 days after the operation. 10,813 genes were detected and quantified in the heart tissues of three groups of rats. Compared with the sham-operated group, 516 genes were differentially expressed in the MI model group. In the DQP-treated group, expressions of 313 of the altered 516 genes in MI model were regulated back towards normal levels. DQP treatment could regulate the altered signaling pathways in ischemic heart tissues and exert an overall regulative effect on multiple targets in signaling pathways.

DQP is composed of *Salvia miltiorrhiza* Bunge and *Panax notoginseng*. Our results showed that DQP had a remarkable cardioprotective effect, as demonstrated by improved LVEF and LVFS. Previous studies have demonstrated that DQP has cardioprotective effects, and the mechanism is related to its regulation on energy metabolism pathways [5, 19, 20]. DQP has also been shown to have anti-inflammatory effect in heart failure model of animals [6]. *Salvia miltiorrhiza* Bunge and *Panax notoginseng* are the major components of DQP, and their effects on cardiovascular diseases have been investigated by numerous studies [21–26]. The major potential effective components of *Salvia miltiorrhiza* Bunge and *Panax notoginseng* include salvianolic acids and *Panax notoginseng* saponins, and they may exert cardioprotective effects through multiple targets in a synergistic way [27, 28]. The effects of DQP on global gene expression patterns in ischemic heart tissues were investigated in this study.

Myocardial ischemic stimulus induced global gene expression changes in the border zone of the heart tissues. In response to a short supply of oxygen and nutrient, several signaling pathways were downregulated, including fatty acid metabolism, amino acid degradation, pyruvate metabolism, PPAR signaling pathway, and citrate cycle. The proteins encoded by these genes were enriched in mitochondria. The top genes with the greatest abundance reductions in the MI model group were enriched in fatty acid metabolism and glycolysis (Figure 2). For example, long-chain acyl-coenzyme A synthetase 1 (ACSL1) could interact with fatty acid transport proteins (FATP) and contribute to the efficient cellular uptake of long-chain fatty acids through vectorial acylation. Inhibition of ACSL1 activity in ischemia heart impairs fatty acid uptake [29]. Acyl-coenzyme A dehydrogenase for medium-chain fatty acids (ACADM) catalyzes the initial step of the mitochondrial fatty acid beta-oxidation pathway [30]. Reduced expressions of these genes suggested that energy metabolism is disrupted in ischemic heart tissues. Among the 177 downregulated genes, DQP treatment upregulated expressions of 101 of these genes, demonstrating that DQP could modulate energy metabolism under ischemic conditions. Our previous study showed that the major components of DQP, salvianolic acids, and *Panax notoginseng* saponins, had effects on energy metabolism [19].

In the MI model group, expressions of 339 genes were upregulated compared with the sham group. According to gene ontology terms, these genes were enriched in response to wounding, extracellular matrix organization, skeletal system development, bone development, regulation of cellular component size, and so forth. Expressions of gene encoding collagens (COL3A1, COL1A1) were greatly increased. Gene encoding natriuretic peptide A (NPPA) implicated in the control of extracellular fluid volume and electrolyte homeostasis was also greatly upregulated. Levels of metalloproteinases (MMP2, MMP23) were upregulated in the model group [31]. These results demonstrated that ventricular remodeling had occurred in response to MI. DQP treatment reversed expressions of these genes towards normal levels. Oxidative stress could produce reactive oxygen species (ROS). ROS could damage endothelial cells and heart tissues, contributing to myocardial fibrosis [32, 33]. Superoxide dismutase (SOD) is a major defense mechanism against ROS [32]. Mitochondrial SOD2 was repressed in ischemic rat models and was upregulated by DQP, suggesting that DQP has a protective antioxidative effect on the heart. Components of DQP, such as salvianolic acid A, salvianolic acid B, ginsenoside Rg1, and so on, have been shown to have antioxidative effects and attenuate hypertrophy [34–37]. Transcription factors involved in inflammation, lipid metabolism, mitochondrial metabolism, and cardiac fibrosis were also regulated back towards normal levels by DQP.

In conclusion, this study demonstrated that DQP could exert cardioprotective effects in the MI model by regulating global gene expression pattern. One limitation of this study is that the sample size was small. The implications of this study warrant further studies with larger sample size to validate the synergistic effects of Chinese medicine. Furthermore, this kind of study will provide insight into the effective components of Chinese medicine and their respective targets in treating complex diseases.

## Figures and Tables

**Table 1:** Nucleotide sequences of primers used in real-time PCR.

Gene	Primers	Lengths (bp)	Temp (°C)
LPL	CGCTCCATCCATCTCTTC	159	49.8
	GGCTCTGACCTTGTTGAT		49.2
ACSL1	GCAGTTCATCGGCATCTT	109	50.6
	GGTTCCAAGCGTGTCATA		49.8
ACADM	ATTACGGAAGAGTTGGCATA	166	54.15
	GTTCTGTCACGCAGTAGG		55.12
ME1	AAGAACCTAGAAGCCATTGT	104	54.23
	GCAGCCATATCCTTGAGAA		54.62
TFB2M	AGAATGCGGATGGAGAGT	141	55.20
	CTGCTGACCAAGGAACTG		55.27
MMP2	AAGTCTGAAGAGTGTGAAGT	180	53.86
	GTGAAGGAGAAGGCTGATT		54.45
ALOX15	CTCAGGCTTGCTACTTCAT	158	54.57
	CTTCTCCATTGTTGCTTCC		53.97
BAX	GATGATTGCTGATGTGGATAC	86	50.50
	AGTTGAAGTTGCCGTCTG		50.40
GPX1	CAATCAGTTCGGACATCAG	133	53.52
	AGCCTTCTCACCATTCAC		54.14
MMP23	GATGGTCCTACAGGTGAAC	195	54.59
	CTGGTCTTGCTGTGAGTG		55.30
GAPDH	TTCAACGGCACAGTCAAG	116	50.70
	TACTCAGCACCAGCATCA		50.50

**Table 2:** Significantly enriched KEGG pathways among upregulated and downregulated genes in ischemic heart. The pathways are ranked according to the order of the increasing *P* values. Gene count referred to the number of genes among all the pathway member genes.

	KEGG pathway ID and name	Gene count	*P* value
Upregulated	rno04610: complement and coagulation cascades	11	4.59*E* − 07
	rno04670: leukocyte transendothelial migration	9	0.001
	rno05322: systemic lupus erythematosus	8	0.001
	rno04510: focal adhesion	11	0.003
	rno04512: ECM-receptor interaction	7	0.004
	rno05200: pathways in cancer	14	0.005
	rno05414: dilated cardiomyopathy	7	0.006

Downregulated	rno00071: fatty acid metabolism	6	7.55*E* − 05
	rno00280: valine, leucine, and isoleucine degradation	6	1.18*E* − 04
	rno00620: pyruvate metabolism	5	7.45*E* − 04
	rno03320: PPAR signaling pathway	6	9.11*E* − 04
	rno00330: arginine and proline metabolism	4	0.018834
	rno00020: citrate cycle (TCA cycle)	3	0.04066
	rno05412: arrhythmogenic right ventricular cardiomyopathy	4	0.044529
	rno00640: propanoate metabolism	3	0.048358
	rno00071: fatty acid metabolism	6	7.55*E* − 05
	rno00280: valine, leucine, and isoleucine degradation	6	1.18*E* − 04

**Table 3:** Top ten significantly enriched GO biological processes among upregulated and downregulated genes in ischemic heart. The GO terms are ranked according to the order of the increasing *P* values. Gene count referred to the number of genes among all the GO member genes.

	Gene ontology ID and terms	Gene count	*P* value
Upregulated	GO:0009611~response to wounding	39	4.65*E* − 16
	GO:0030198~extracellular matrix organization	16	6.64*E* − 11
	GO:0001501~skeletal system development	24	7.24*E* − 10
	GO:0060348~bone development	17	1.11*E* − 09
	GO:0032535~regulation of cellular component size	22	1.92*E* − 09
	GO:0001503~ossification	16	2.21*E* − 09
	GO:0001558~regulation of cell growth	19	2.34*E* − 09
	GO:0040008~regulation of growth	24	7.57*E* − 09
	GO:0043062~extracellular structure organization	16	4.00*E* − 08
	GO:0008361~regulation of cell size	18	6.08*E* − 08

Downregulated	GO:0006091~generation of precursor metabolites and energy	12	1.60*E* − 05
	GO:0044275~cellular carbohydrate catabolic process	7	6.08*E* − 05
	GO:0006007~glucose catabolic process	6	2.01*E* − 04
	GO:0055114~oxidation reduction	17	2.14*E* − 04
	GO:0046365~monosaccharide catabolic process	6	2.37*E* − 04
	GO:0019320~hexose catabolic process	6	2.37*E* − 04
	GO:0046395~carboxylic acid catabolic process	7	3.17*E* − 04
	GO:0016054~organic acid catabolic process	7	3.17*E* − 04
	GO:0016052~carbohydrate catabolic process	7	3.36*E* − 04
	GO:0043648~dicarboxylic acid metabolic process	5	4.94*E* − 04

**Table 4:** Enriched downregulated pathways and biological processes by DQP treatment.

Term	%	*P* value	Genes
KEGG pathway			
*Staphylococcus aureus* infection	3.6	0.000015	C1QA, C1QB, C5AR1, LOC498276, CFH, RT1-DMA, C1QC
Phagosome	4.1	0.003	ACTB, RAB5C, NCF4, LOC498276, SCARB1, RT1-DMA, TUBA1C, SEC61A1
Complement and coagulation cascades	2.6	0.006	C1QA, C1QB, C5AR1, CFH, C1QC
African trypanosomiasis	2.1	0.006	VCAM1, F2RL1, LOC100134871, HBB
Leukocyte transendothelial migration	3.1	0.007	ACTB, VCAM1, MYL7, NCF4, CLDN5, MMP2
Tuberculosis	3.6	0.010	LSP1, RAB5C, LOC498276, TGFB3, FCER1G, RT1-DMA, LBP
Platelet activation	3.1	0.011	ACTB, GP1BB, LOC498276, COL3A1, FCER1G, COL5A2
ECM-receptor interaction	2.6	0.012	GP1BB, COL3A1, COL5A2, SPP1, FN1
Glutathione metabolism	2.1	0.020	GSTA3, GPX3, GSTT1, GPX7
Malaria	2.1	0.021	VCAM1, LOC100134871, TGFB3, HBB

GO_biological process			
Cell adhesion	6.7	0.000017	IGFBP7, COL16A1, VCAM1, WISP2, CTGF, GP1BB, FBLN5, VCAN, GPNMB, SPON1, FN1, AOC3, SPP1
Regulation of cell growth	3.6	0.000045	WISP2, CREB3, CTGF, IGFBP7, FBLN5, IGFBP6, IGFBP4
Aging	6.2	0.00042	VCAM1, C1QB, GSTA3, LITAF, CTGF, ELN, COL3A1, TGFB3, TIMP2, TIMP3, MMP2, AOC3
Collagen fibril organization	2.6	0.001	COL3A1, LOXL2, COL5A2, ANXA2, DPT
Positive regulation of fibroblast proliferation	3.1	0.001	FBLN1, TGIF1, AQP1, MYC, ANXA2, FN1
Integrin-mediated signaling pathway	3.1	0.002	FBLN1, CTGF, ADAMTS15, COL3A1, FCER1G, TYROBP
Elastic fiber assembly	1.5	0.003	FBLN5, ELN, MFAP4
Neutrophil chemotaxis	2.6	0.003	C5AR1, LGALS3, FCER1G, CCL19, SPP1
Extracellular matrix organization	3.1	0.003	FBLN1, LGALS3, FBLN5, ELN, CCDC80, FN1
Ossification	3.1	0.003	ALOX15, CTGF, MGP, COL5A2, SPP1, FN1

**Table 5:** Enriched upregulated pathways and biological processes by DQP treatment.

Term	%	*P* value	Genes
KEGG pathway			
Carbon metabolism	7.5	0.000027	ME1, ALDOA, GOT2, ME3, ACADM, ENO3, SUCLA2
Biosynthesis of antibiotics	6.5	0.004	ALDOA, GOT2, ACADM, ENO3, SUCLA2, HADHB
Metabolic pathways	15.1	0.008	NDUFA4, ALDOA, ME1, ME3, ACADM, CHKB, UQCRFS1, HADHB, GOT2, ACSL1, CKM, MCCC1, ENO3, SUCLA2
Fatty acid degradation	3.2	0.023	ACSL1, ACADM, HADHB
Fatty acid metabolism	3.2	0.030	ACSL1, ACADM, HADHB
Valine, leucine, and isoleucine degradation	3.2	0.031	ACADM, MCCC1, HADHB

GO_biological process			
Pyruvate metabolic process	3.2	0.001	ME1, ME3, PFKFB2
Response to hormone	5.4	0.001	ME1, BDNF, ACADM, ADRA1B, UQCRFS1
Response to drug	8.6	0.013	BDNF, ACSL1, ACADM, ADRA1B, ENO3, AQP7, UQCRFS1, SOD2
Glycolytic process	3.2	0.015	ALDOA, PFKFB2, ENO3
Negative regulation of fat cell differentiation	3.2	0.021	VEGFA, INSIG1, SOD2
Regulation of synaptic plasticity	3.2	0.021	BDNF, PSEN2, RAPGEF2
Muscle contraction	3.2	0.024	TRDN, MYOM2, TMOD4
Response to cold	3.2	0.026	ACADM, VEGFA, SOD2
Intraciliary transport involved in cilium morphogenesis	2.2	0.033	IFT81, PCM1
Oxidation-reduction process	8.6	0.036	ME1, NDUFA4, ME3, ACADM, L2HGDH, UQCRFS1, OXR1, HADHB

## Supplementary Materials

Supplementary Materials Supplemental Table 1: basic information of RNA-sequencing data.
