# Supplementary material for: Multitarget Effects of Danqi Pill on Global Gene Expression Changes in Myocardial Ischemia
Source: Int J Genomics. 2018 Feb 1;2018:9469670. doi: 10.1155/2018/9469670 (PMC5816862; doi:10.1155/2018/9469670)
Supplement: Supplementary Materials — Supplemental Table 1: basic information of RNA-sequencing data. [file 9469670.f1.docx]

Supplemental table1: Basic information of RNA sequencing data

| Sample | Total Tags | Mapped to Gene Tags | Mapped to Gene Tags | Unambiguous Tag-mapped Genes |
| --- | --- | --- | --- | --- |
| DQP1 | 3809596 | 1830130 | 48.04% | 9234 |
| DQP2 | 3788401 | 2026037 | 53.48% | 9537 |
| DQP3 | 3817730 | 2052412 | 53.76% | 9424 |
| Sham1 | 3838953 | 1856134 | 48.35% | 8810 |
| Sham2 | 3832123 | 1806846 | 47.15% | 8907 |
| Sham3 | 3824847 | 1835927 | 48.00% | 8795 |
| Model1 | 3801073 | 1875830 | 49.35% | 9322 |
| Model2 | 3817508 | 2171399 | 56.88% | 9344 |
| Model3 | 3828761 | 2035489 | 53.16% | 9138 |
